# Supplementary material for: DeepLabStream enables closed-loop behavioral experiments using deep learning-based markerless, real-time posture detection
Source: Commun Biol. 2021 Jan 29;4:130. doi: 10.1038/s42003-021-01654-9 (PMC7846585; doi:10.1038/s42003-021-01654-9)
Supplement: Supplementary file 2 — Supplementary Information [file 42003_2021_1654_MOESM2_ESM.pdf]

Supplementary Material

DeepLabStream enables closed-loop behavioral experiments using deep learning-based markerless, real-time posture detection

Jens F. Schweihoff [1], Matvey Loshakov [1], Irina Pavlova [1], Laura Kück [2], Laura A. Ewell [2] and Martin K. Schwarz [1*]

[1] Functional Neuroconnectomics Group, Institute of Experimental Epileptology and Cognition Research, Medical Faculty, University of Bonn, Bonn, Germany.

[2] Institute of Experimental Epileptology and Cognition Research, Medical Faculty, University of Bonn, Bonn, Germany.

* Correspondence: Martin.Schwarz@ukbonn.de

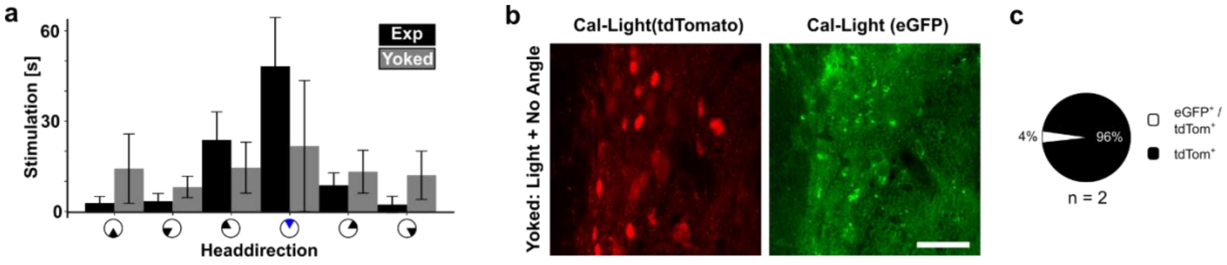

Supplementary Figure 1. Optogenetic labeling of head direction dependent activity in neurons: Yoked Group. a, Average light stimulation in both experimental and yoked group during each session as a function of head direction (60° bins) with target window (blue wedge) indicating the DLStream triggered stimulation onset angles. Exp: n = 10 mice, black bars; Yoked: n = 8 mice, grey bars. Error bars represent standard deviation. Experimental and yoked groups have the same total stimulation time, but the distribution differs such that yoked group has approximately equal stimulation times across varying head direction angles. **b**, Close up (similar region of interest as shown in Fig. 4g) of representative expression in mice from the yoked group that was stimulated based on the stimulus times taken from a previous session of a paired experimental animal. Left: tdTomato expression (red) indicating expression of Cal-Light viruses. Right: Light induced eGFP expression (green). The bar represents 50 µm. **c**, Ratio between infected neurons ($tdTom^+$) and activity dependent labelled neurons ($eGFP^+/tdTom^+$) in mice matching selection criteria (see Methods) in the yoked group. n = 2 mice. Light stimulation of the same duration as in the experimental group but not the same head direction specificity was not enough to reliably activate the Cal-Light labeling system, suggesting that the resulting coincidence between activity during light stimulation of the neurons was not high/often enough to result in a sufficient number of light stimulation that coincided with the neurons activity, as the head direction dependency of the stimulation was not given.

a

Group	Number of Animals	Match	Mismatch
Exp	10	2	8
Yoked	8	2	6
NoLight	2	1	1
Total	20	5	15

b

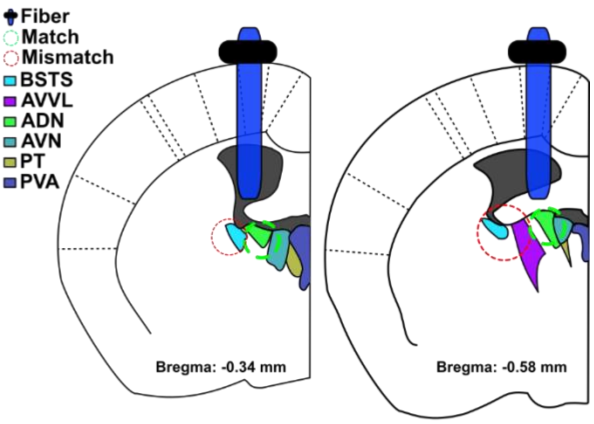

c

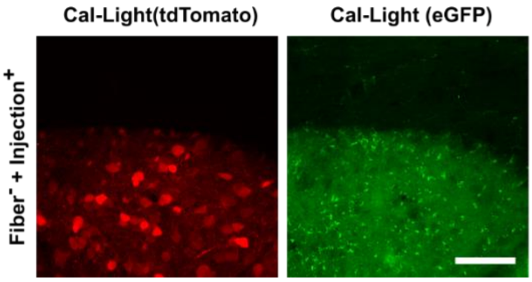

Supplementary Figure 2. Quantification of optogenetic labeling of head direction dependent activity in neurons. a, Table of all injected and implanted animals divided into experimental groups and success categories. ‘Match’ occurred when the viral injection was successfully targeted to the ADN and optic fiber was placed above the ADN. Tissue processed from the ‘match’ case were used for quantification of labeled neurons. ‘Mismatch’ occurs either when the viral injection or fiber placement missed the ADN. **b**, Schematic representation of injection sites. When the ADN was missed, injections were too lateral, hitting either the BSTS or AVVL. The blue ferrule represents optimal placement. **c**, Close up (similar region of interest as shown in Fig. 4g) of representative expression in mice with incorrect fiber placement. Left: tdTomato expression (red) indicating expression of Cal-Light viruses. Right: Activity dependent and light induced eGFP expression (green). The bar represents 50 µm. BSTS: bed nucleus of stria terminalis, supracapsular part; AVVL: anteroventral thalamic nucleus, ventrolateral part; ADN: anterodorsal thalamic nucleus; AVN: anteroventral thalamic nucleus; PT: paratenial thalamic nucleus; PVA: paraventricular thalamic nucleus, anterior part.

Supplementary Table 1. Performance of different network architectures in DLStream in relation to number of estimated body parts and image resolution.

		3 Body parts	*9 Body parts*	*13 Body parts*
Network	**Resolution**	**Average FPS**	**Average FPS**	**Average FPS**
MobileNetv2	**320x256**	**164.04 +/- 7.28**	**130.55 +/- 6.51**	**79.29 +/- 19.18**
	416x341	119.73 +/- 8.42	86.64 +/- 3.43	67.50 +/- 10.95
	640x512	60.51 +/- 2.01	54.24 +/- 0.94	46.76 +/- 2.91
	1280x1024	16.61 +/- 0.26	16.19 +/- 0.20	14.99 +/- 1.14
ResNet50	320x256	107.58 +/- 8.68	94.30 +/- 6.21	67.01 +/- 10.36
	416x341	79.52 +/- 3.00	66.70 +/- 1.83	55.49 +/- 4.99
	640x512	44.92 +/- 1.44	41.03 +/- 0.52	36.50 +/- 1.88
	1280x1024	13.61 +/- 0.35	13.25 +/- 0.12	12.32 +/- 0.86
ResNet101	320x256	68.29 +/- 2.23	64.72 +/- 1.86	60.35 +/- 6.13
	416x341	54.85 +/- 1.51	49.99 +/- 0.94	48.34 +/- 3.01
	640x512	32.05 +/- 0.51	30.47 +/- 0.33	30.18 +/- 1.88
	1280x1024	9.80 +/- 0.28	10.33 +/- 0.34	9.92 +/- 0.76

a

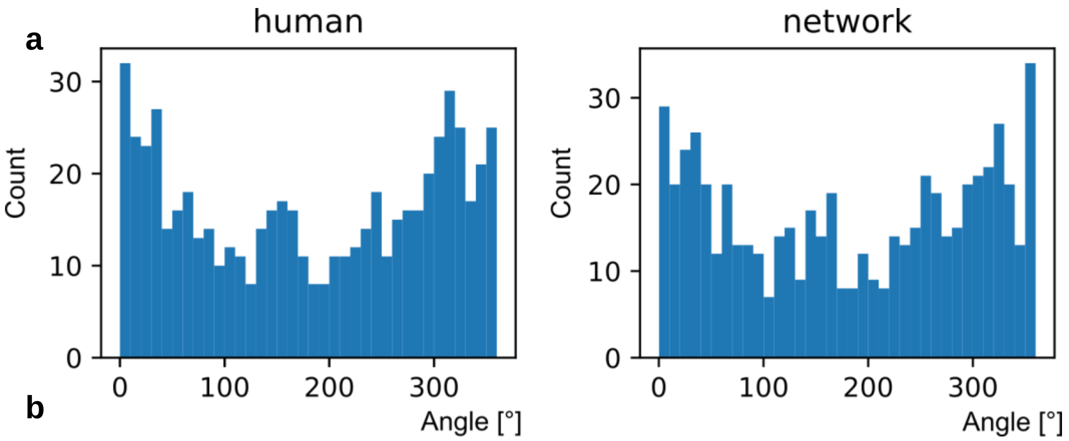

b

Window size [°]	False positive detection [%]	False negative detection [%]
**60**	11.6 ± 4.8	11.1 ± 4.1
**50**	13.2 ± 5.3	13.7 ± 5.4
**40**	14.7 ± 5.8	14.7 ± 5.9
**30**	20.1 ± 11.2	19.8 ± 10.5
**20**	29.0 ± 20.7	28.2 ± 18.7
**10**	72.8 ± 75.6	75.8 ± 89.0

Supplementary Figure 3. Estimation of accuracy of head direction triggers with different angle window sizes.

a, Histograms (10° bins between 0-360°) of the distribution of the labeled dataset (n = 597), with human annotation (right) and head direction angle based on network pose estimation (right) using the network trained for the optogenetic stimulation task. **b**, Table showing the false positive and false negative detection rate of the network pose estimation against human annotation in several differently sized angle windows (theoretical triggers). To counter any effects of non-uniform distribution, the window was moved around in steps and the average, as well as the standard deviation was taken from all detected events. An event was counted as false positive if the pose estimation resulted in a head direction within the window, while the human annotation did not (and vice versa for false negative).

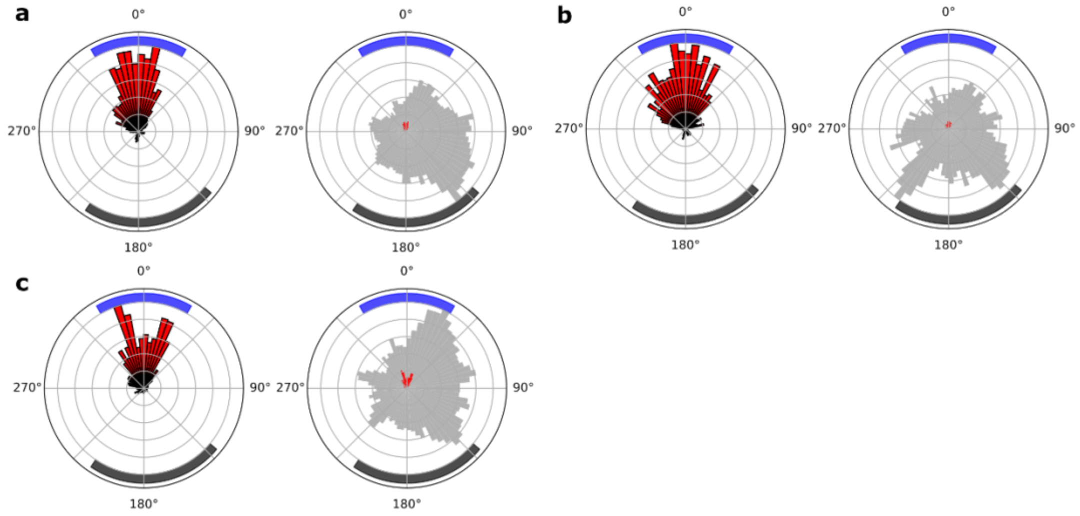

Supplementary Figure 4. Examples of head direction angles during optogenetic light stimulation.

a-b, Left: Example radial histogram of all head directions (5° bins) during stimulation (red) within one session (normalized to the maximum value). Right: Radial histogram of all head directions during the whole session (grey) and during stimulation (red) (normalized to the maximum value of the entire session). Rings represent quantiles in 20 % steps. Each panel shows a session from a different mouse. **c,** Example radial histogram of all head directions (same representation as in **a-b**) from the same mouse shown in **a** in the next session. Note that the mouse is showing different distributions of head direction between sessions in both the stimulation events and the overall session, while the stimulation is mostly limited to the angle window (thick blue arc)
