# Supplementary material for: DeepLabStream enables closed-loop behavioral experiments using deep learning-based markerless, real-time posture detection
Source: Commun Biol. 2021 Jan 29;4:130. doi: 10.1038/s42003-021-01654-9 (PMC7846585; doi:10.1038/s42003-021-01654-9)
Supplement: Supplementary file 3 — Description of Supplementary Files [file 42003_2021_1654_MOESM3_ESM.pdf]

Description of Additional Supplementary Files

File name: Supplementary Movie 1 | Example video of speed trigger.

Description: The movie shows an example implementation of a posture sequence detection trigger (speed) that is configured to detect the mouse's movement speed greater than 10 px (based on tail root pose estimation) within 5 consecutive frames. If the animal is moving faster than a user defined threshold within the set time window, the stimulation (blue light, middle right) is activated. Additionally, DLStream reports the current frame number (top left, red), current time and fps (bottom right, blue) and current state of the trigger (mouse's nose, red/green text). The trigger was set to a low threshold to detect any meaningful movement of the main body (like walking/running) but can be set to only detect episodes of fast running by adjusting the threshold. A similar "freeze" trigger that does the reverse is available in our code as well.

File name: Supplementary Data 1 | Source data regarding preference task.

Description: Source data represented in the bar plot in Figure 3 d of the main manuscript. Odor locations are named by the odor presented to the mouse (Rose, Vanillin, Valeric Acid, and Acetophenon). Each mouse had up to two trials (see Methods and Fig. 3 for further details). The average and standard deviation for each odor location, as well as the results of a one-tailed, paired t-test are shown below the table.

File name: Supplementary Data 2 | Source data regarding optogenetic stimulation task.

Description: The analyzed source data (processed DLStream output) represented in the plots in Figure 4 c, d, e, f of the main manuscript. The radial histograms (c, d) represent the data collected from the "Analysis _angle_neck_nose_point" column throughout the whole experiment. The 3D plot (e), as well as the heatmaps (f) are taken from the columns "Animal1_tailroot_x" and "Animal1_tailroot_y" describing the position of the mouse during the experiment in relation to the stimulation events in the columns "Experiment_trial" (describing if the stimulation is running as a bool) and "Experiment_trial_label" (identifying the event ID).

File name: Supplementary Data 3 | Source data regarding angular distribution in optogenetic stimulation task.

Description: The binned bar plot/histogram source data (processed DLStream output) represented in the plot in Figure 4i of the main manuscript. The source data describes the head direction angle (binned) for each session by each mouse during stimulation.
